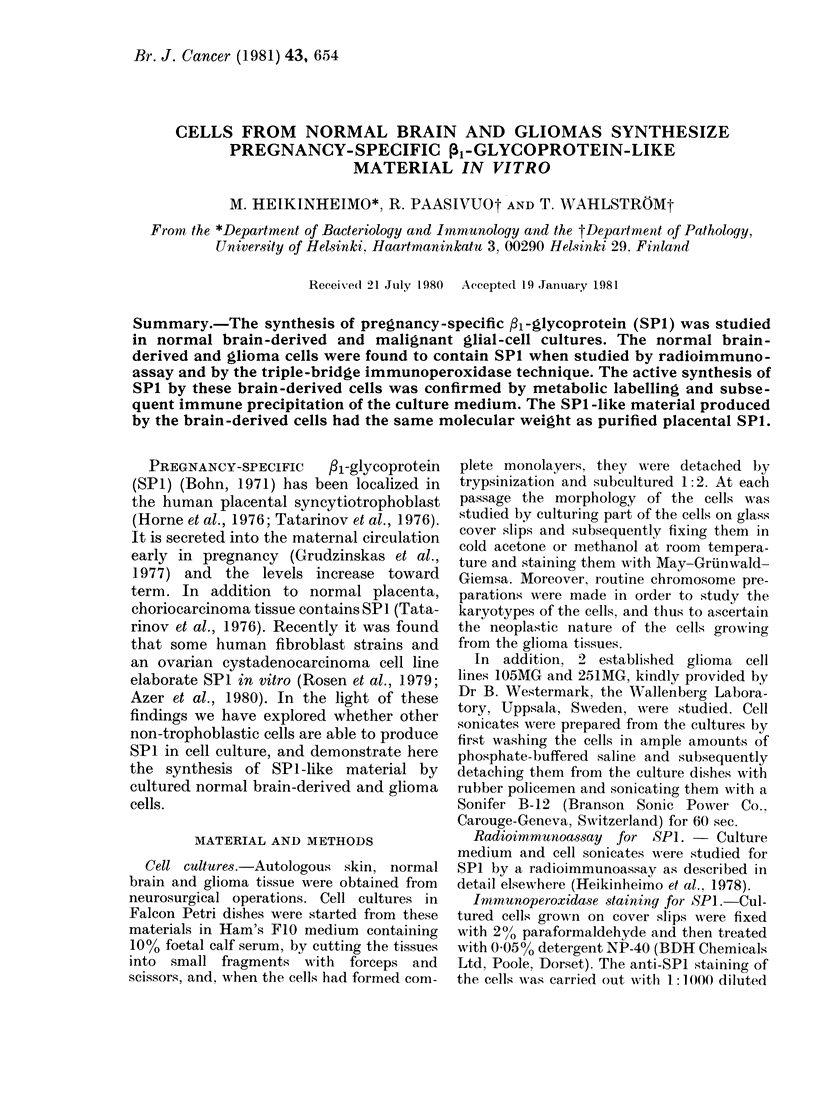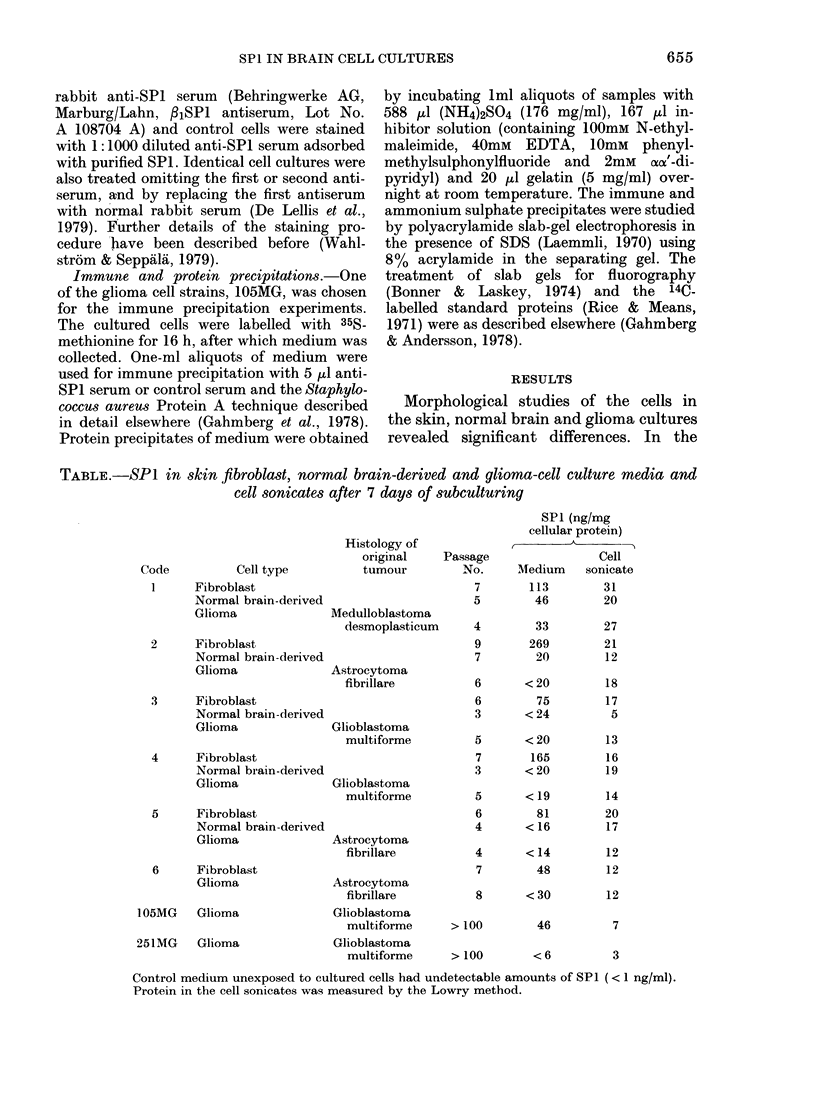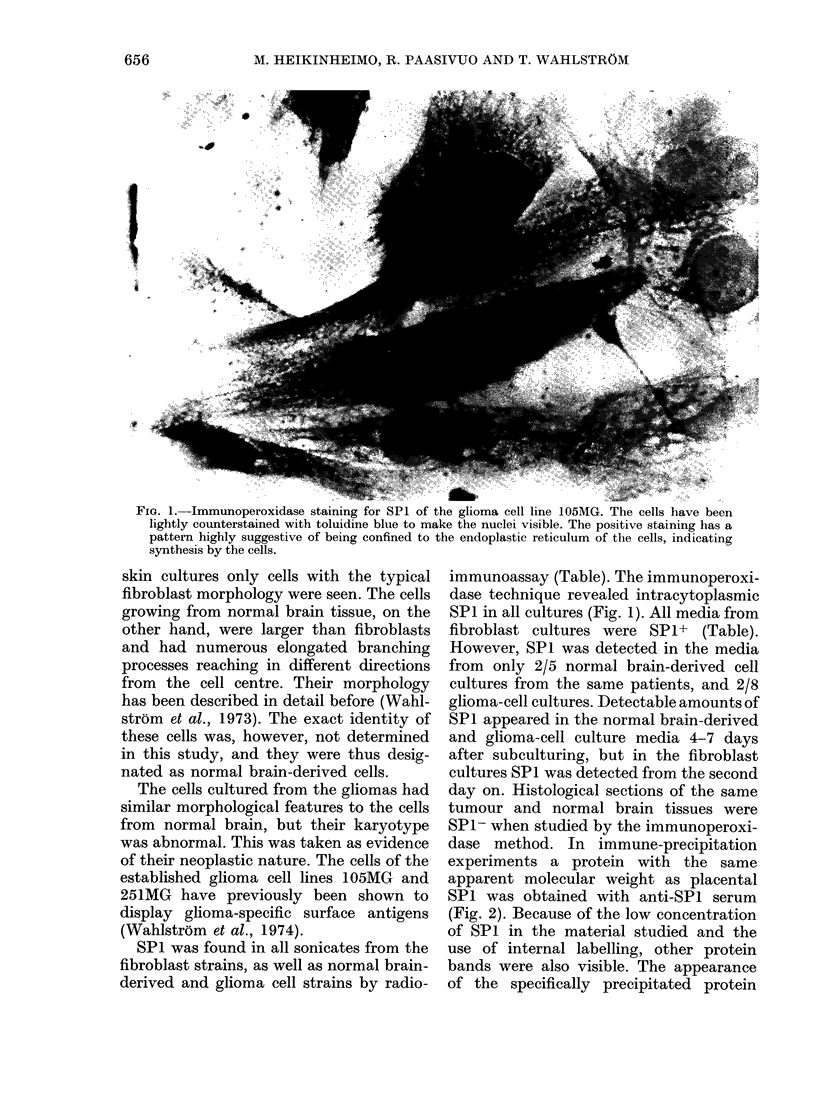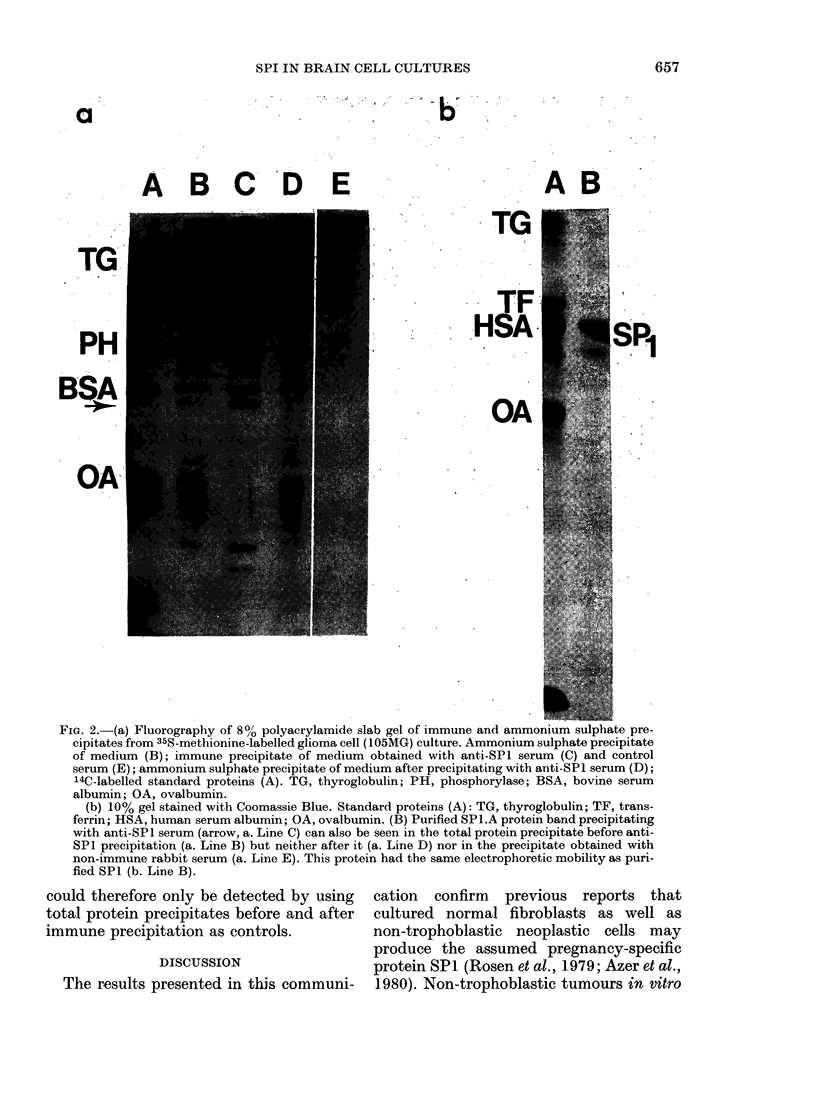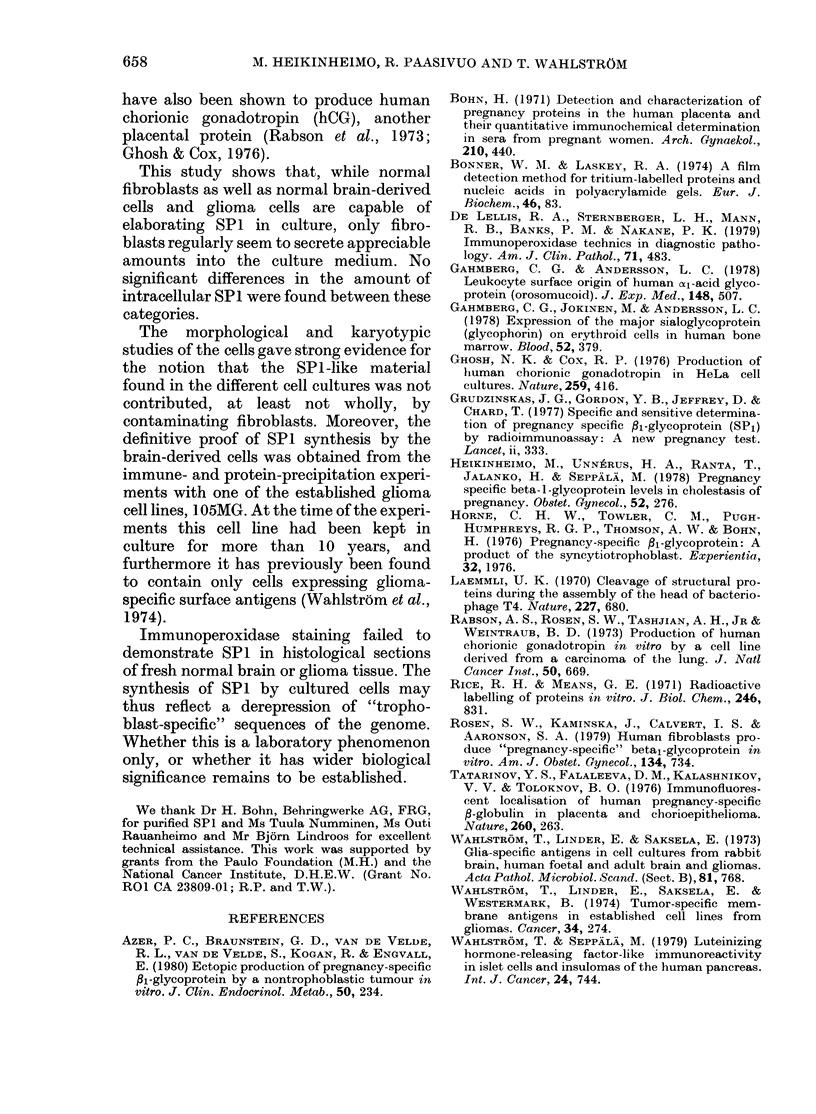# Cells from normal brain and gliomas synthesize pregnancy-specific beta 1-glycoprotein-like material in vitro.

**DOI:** 10.1038/bjc.1981.96

**Published:** 1981-05

**Authors:** M. Heikinheimo, R. Paasivuo, T. Wahlström

## Abstract

**Images:**


*Br. J. Cancer* (1981) **43**, 654

### CELLS FROM NORMAL BRAIN AND GLIOMAS SYNTHESIZE PREGNANCY-SPECIFIC $\beta_1$-GLYCOPROTEIN-LIKE MATERIAL *IN VITRO*

M. HEIKINHEIMO*, R. PAASIVUO† AND T. WAHLSTRÖM†

*From the *Department of Bacteriology and Immunology and the †Department of Pathology, University of Helsinki, Haartmaninkatu* 3, 00290 *Helsinki* 29, *Finland*

Received 21 July 1980 Accepted 19 January 1981

**Summary.—The synthesis of pregnancy-specific $\beta_1$-glycoprotein (SP1) was studied in normal brain-derived and malignant glial-cell cultures. The normal brain-derived and glioma cells were found to contain SP1 when studied by radioimmunoassay and by the triple-bridge immunoperoxidase technique. The active synthesis of SP1 by these brain-derived cells was confirmed by metabolic labelling and subsequent immune precipitation of the culture medium. The SP1-like material produced by the brain-derived cells had the same molecular weight as purified placental SP1.**

PREGNANCY-SPECIFIC $\beta_1$-glycoprotein (SP1) (Bohn, 1971) has been localized in the human placental syncytiotrophoblast (Horne *et al.*, 1976; Tatarinov *et al.*, 1976). It is secreted into the maternal circulation early in pregnancy (Grudzinskas *et al.*, 1977) and the levels increase toward term. In addition to normal placenta, choriocarcinoma tissue contains SP1 (Tatarinov *et al.*, 1976). Recently it was found that some human fibroblast strains and an ovarian cystadenocarcinoma cell line elaborate SP1 *in vitro* (Rosen *et al.*, 1979; Azer *et al.*, 1980). In the light of these findings we have explored whether other non-trophoblastic cells are able to produce SP1 in cell culture, and demonstrate here the synthesis of SP1-like material by cultured normal brain-derived and glioma cells.

### MATERIAL AND METHODS

*Cell cultures.*—Autologous skin, normal brain and glioma tissue were obtained from neurosurgical operations. Cell cultures in Falcon Petri dishes were started from these materials in Ham's F10 medium containing 10% foetal calf serum, by cutting the tissues into small fragments with forceps and scissors, and, when the cells had formed complete monolayers, they were detached by trypsinization and subcultured 1:2. At each passage the morphology of the cells was studied by culturing part of the cells on glass cover slips and subsequently fixing them in cold acetone or methanol at room temperature and staining them with May–Grünwald–Giemsa. Moreover, routine chromosome preparations were made in order to study the karyotypes of the cells, and thus to ascertain the neoplastic nature of the cells growing from the glioma tissues.

In addition, 2 established glioma cell lines 105MG and 251MG, kindly provided by Dr B. Westermark, the Wallenberg Laboratory, Uppsala, Sweden, were studied. Cell sonicates were prepared from the cultures by first washing the cells in ample amounts of phosphate-buffered saline and subsequently detaching them from the culture dishes with rubber policemen and sonicating them with a Sonifer B-12 (Branson Sonic Power Co., Carouge-Geneva, Switzerland) for 60 sec.

*Radioimmunoassay for SP*1. — Culture medium and cell sonicates were studied for SP1 by a radioimmunoassay as described in detail elsewhere (Heikinheimo *et al.*, 1978).

*Immunoperoxidase staining for SP*1.—Cultured cells grown on cover slips were fixed with 2% paraformaldehyde and then treated with 0·05% detergent NP-40 (BDH Chemicals Ltd, Poole, Dorset). The anti-SP1 staining of the cells was carried out with 1:1000 diluted rabbit anti-SP1 serum (Behringwerke AG, Marburg/Lahn, $\beta_1$SP1 antiserum, Lot No. A 108704 A) and control cells were stained with 1:1000 diluted anti-SP1 serum adsorbed with purified SP1. Identical cell cultures were also treated omitting the first or second antiserum, and by replacing the first antiserum with normal rabbit serum (De Lellis *et al.*, 1979). Further details of the staining procedure have been described before (Wahlström & Seppälä, 1979).

*Immune and protein precipitations.*—One of the glioma cell strains, 105MG, was chosen for the immune precipitation experiments. The cultured cells were labelled with $^{35}$S-methionine for 16 h, after which medium was collected. One-ml aliquots of medium were used for immune precipitation with 5 $\mu$l anti-SP1 serum or control serum and the *Staphylococcus aureus* Protein A technique described in detail elsewhere (Gahmberg *et al.*, 1978). Protein precipitates of medium were obtained by incubating 1ml aliquots of samples with 588 $\mu$l $(NH_4)_2SO_4$ (176 mg/ml), 167 $\mu$l inhibitor solution (containing 100mM N-ethylmaleimide, 40mM EDTA, 10mM phenylmethylsulphonylfluoride and 2mM αα'-dipyridyl) and 20 $\mu$l gelatin (5 mg/ml) overnight at room temperature. The immune and ammonium sulphate precipitates were studied by polyacrylamide slab-gel electrophoresis in the presence of SDS (Laemmli, 1970) using 8% acrylamide in the separating gel. The treatment of slab gels for fluorography (Bonner & Laskey, 1974) and the $^{14}$C-labelled standard proteins (Rice & Means, 1971) were as described elsewhere (Gahmberg & Andersson, 1978).

### RESULTS

Morphological studies of the cells in the skin, normal brain and glioma cultures revealed significant differences. In the

TABLE.—*SP1 in skin fibroblast, normal brain-derived and glioma-cell culture media and cell sonicates after 7 days of subculturing*

| Code | Cell type | Histology of original tumour | Passage No. | SP1 (ng/mg cellular protein) Medium | Cell sonicate |
|---|---|---|---|---|---|
| 1 | Fibroblast | | 7 | 113 | 31 |
| | Normal brain-derived | | 5 | 46 | 20 |
| | Glioma | Medulloblastoma desmoplasticum | 4 | 33 | 27 |
| 2 | Fibroblast | | 9 | 269 | 21 |
| | Normal brain-derived | | 7 | 20 | 12 |
| | Glioma | Astrocytoma fibrillare | 6 | < 20 | 18 |
| 3 | Fibroblast | | 6 | 75 | 17 |
| | Normal brain-derived | | 3 | < 24 | 5 |
| | Glioma | Glioblastoma multiforme | 5 | < 20 | 13 |
| 4 | Fibroblast | | 7 | 165 | 16 |
| | Normal brain-derived | | 3 | < 20 | 19 |
| | Glioma | Glioblastoma multiforme | 5 | < 19 | 14 |
| 5 | Fibroblast | | 6 | 81 | 20 |
| | Normal brain-derived | | 4 | < 16 | 17 |
| | Glioma | Astrocytoma fibrillare | 4 | < 14 | 12 |
| 6 | Fibroblast | | 7 | 48 | 12 |
| | Glioma | Astrocytoma fibrillare | 8 | < 30 | 12 |
| 105MG | Glioma | Glioblastoma multiforme | > 100 | 46 | 7 |
| 251MG | Glioma | Glioblastoma multiforme | > 100 | < 6 | 3 |

Control medium unexposed to cultured cells had undetectable amounts of SP1 (< 1 ng/ml). Protein in the cell sonicates was measured by the Lowry method.

FIG. 1.—Immunoperoxidase staining for SP1 of the glioma cell line 105MG. The cells have been lightly counterstained with toluidine blue to make the nuclei visible. The positive staining has a pattern highly suggestive of being confined to the endoplastic reticulum of the cells, indicating synthesis by the cells.

skin cultures only cells with the typical fibroblast morphology were seen. The cells growing from normal brain tissue, on the other hand, were larger than fibroblasts and had numerous elongated branching processes reaching in different directions from the cell centre. Their morphology has been described in detail before (Wahlström *et al.*, 1973). The exact identity of these cells was, however, not determined in this study, and they were thus designated as normal brain-derived cells.

The cells cultured from the gliomas had similar morphological features to the cells from normal brain, but their karyotype was abnormal. This was taken as evidence of their neoplastic nature. The cells of the established glioma cell lines 105MG and 251MG have previously been shown to display glioma-specific surface antigens (Wahlström *et al.*, 1974).

SP1 was found in all sonicates from the fibroblast strains, as well as normal brain-derived and glioma cell strains by radioimmunoassay (Table). The immunoperoxidase technique revealed intracytoplasmic SP1 in all cultures (Fig. 1). All media from fibroblast cultures were $SP1^+$ (Table). However, SP1 was detected in the media from only 2/5 normal brain-derived cell cultures from the same patients, and 2/8 glioma-cell cultures. Detectable amounts of SP1 appeared in the normal brain-derived and glioma-cell culture media 4–7 days after subculturing, but in the fibroblast cultures SP1 was detected from the second day on. Histological sections of the same tumour and normal brain tissues were $SP1^-$ when studied by the immunoperoxidase method. In immune-precipitation experiments a protein with the same apparent molecular weight as placental SP1 was obtained with anti-SP1 serum (Fig. 2). Because of the low concentration of SP1 in the material studied and the use of internal labelling, other protein bands were also visible. The appearance of the specifically precipitated protein

FIG. 2.—(a) Fluorography of 8% polyacrylamide slab gel of immune and ammonium sulphate precipitates from $^{35}$S-methionine-labelled glioma cell (105MG) culture. Ammonium sulphate precipitate of medium (B); immune precipitate of medium obtained with anti-SP1 serum (C) and control serum (E); ammonium sulphate precipitate of medium after precipitating with anti-SP1 serum (D); $^{14}$C-labelled standard proteins (A). TG, thyroglobulin; PH, phosphorylase; BSA, bovine serum albumin; OA, ovalbumin.

(b) 10% gel stained with Coomassie Blue. Standard proteins (A): TG, thyroglobulin; TF, transferrin; HSA, human serum albumin; OA, ovalbumin. (B) Purified SP1.A protein band precipitating with anti-SP1 serum (arrow, a. Line C) can also be seen in the total protein precipitate before anti-SP1 precipitation (a. Line B) but neither after it (a. Line D) nor in the precipitate obtained with non-immune rabbit serum (a. Line E). This protein had the same electrophoretic mobility as purified SP1 (b. Line B).

could therefore only be detected by using total protein precipitates before and after immune precipitation as controls.

### DISCUSSION

The results presented in this communication confirm previous reports that cultured normal fibroblasts as well as non-trophoblastic neoplastic cells may produce the assumed pregnancy-specific protein SP1 (Rosen *et al.*, 1979; Azer *et al.*, 1980). Non-trophoblastic tumours *in vitro*

have also been shown to produce human chorionic gonadotropin (hCG), another placental protein (Rabson *et al.*, 1973; Ghosh & Cox, 1976).

This study shows that, while normal fibroblasts as well as normal brain-derived cells and glioma cells are capable of elaborating SP1 in culture, only fibroblasts regularly seem to secrete appreciable amounts into the culture medium. No significant differences in the amount of intracellular SP1 were found between these categories.

The morphological and karyotypic studies of the cells gave strong evidence for the notion that the SP1-like material found in the different cell cultures was not contributed, at least not wholly, by contaminating fibroblasts. Moreover, the definitive proof of SP1 synthesis by the brain-derived cells was obtained from the immune- and protein-precipitation experiments with one of the established glioma cell lines, 105MG. At the time of the experiments this cell line had been kept in culture for more than 10 years, and furthermore it has previously been found to contain only cells expressing glioma-specific surface antigens (Wahlström *et al.*, 1974).

Immunoperoxidase staining failed to demonstrate SP1 in histological sections of fresh normal brain or glioma tissue. The synthesis of SP1 by cultured cells may thus reflect a derepression of "trophoblast-specific" sequences of the genome. Whether this is a laboratory phenomenon only, or whether it has wider biological significance remains to be established.

We thank Dr H. Bohn, Behringwerke AG, FRG, for purified SP1 and Ms Tuula Numminen, Ms Outi Rauanheimo and Mr Björn Lindroos for excellent technical assistance. This work was supported by grants from the Paulo Foundation (M.H.) and the National Cancer Institute, D.H.E.W. (Grant No. RO1 CA 23809-01; R.P. and T.W.).

### REFERENCES

AZER, P. C., BRAUNSTEIN, G. D., VAN DE VELDE, R. L., VAN DE VELDE, S., KOGAN, R. & ENGVALL, E. (1980) Ectopic production of pregnancy-specific $\beta_1$-glycoprotein by a nontrophoblastic tumour *in vitro*. *J. Clin. Endocrinol. Metab.*, **50**, 234.

BOHN, H. (1971) Detection and characterization of pregnancy proteins in the human placenta and their quantitative immunochemical determination in sera from pregnant women. *Arch. Gynaekol.*, **210**, 440.

BONNER, W. M. & LASKEY, R. A. (1974) A film detection method for tritium-labelled proteins and nucleic acids in polyacrylamide gels. *Eur. J. Biochem.*, **46**, 83.

DE LELLIS, R. A., STERNBERGER, L. H., MANN, R. B., BANKS, P. M. & NAKANE, P. K. (1979) Immunoperoxidase technics in diagnostic pathology. *Am. J. Clin. Pathol.*, **71**, 483.

GAHMBERG, C. G. & ANDERSSON, L. C. (1978) Leukocyte surface origin of human $\alpha_1$-acid glycoprotein (orosomucoid). *J. Exp. Med.*, **148**, 507.

GAHMBERG, C. G., JOKINEN, M. & ANDERSSON, L. C. (1978) Expression of the major sialoglycoprotein (glycophorin) on erythroid cells in human bone marrow. *Blood*, **52**, 379.

GHOSH, N. K. & COX, R. P. (1976) Production of human chorionic gonadotropin in HeLa cell cultures. *Nature*, **259**, 416.

GRUDZINSKAS, J. G., GORDON, Y. B., JEFFREY, D. & CHARD, T. (1977) Specific and sensitive determination of pregnancy specific $\beta_1$-glycoprotein ($SP_1$) by radioimmunoassay: A new pregnancy test. *Lancet*, ii, 333.

HEIKINHEIMO, M., UNNÉRUS, H. A., RANTA, T., JALANKO, H. & SEPPÄLÄ, M. (1978) Pregnancy specific beta-1-glycoprotein levels in cholestasis of pregnancy. *Obstet. Gynecol.*, **52**, 276.

HORNE, C. H. W., TOWLER, C. M., PUGH-HUMPHREYS, R. G. P., THOMSON, A. W. & BOHN, H. (1976) Pregnancy-specific $\beta_1$-glycoprotein: A product of the syncytiotrophoblast. *Experientia*, **32**, 1976.

LAEMMLI, U. K. (1970) Cleavage of structural proteins during the assembly of the head of bacteriophage T4. *Nature*, **227**, 680.

RABSON, A. S., ROSEN, S. W., TASHJIAN, A. H., JR & WEINTRAUB, B. D. (1973) Production of human chorionic gonadotropin *in vitro* by a cell line derived from a carcinoma of the lung. *J. Natl Cancer Inst.*, **50**, 669.

RICE, R. H. & MEANS, G. E. (1971) Radioactive labelling of proteins *in vitro*. *J. Biol. Chem.*, **246**, 831.

ROSEN, S. W., KAMINSKA, J., CALVERT, I. S. & AARONSON, S. A. (1979) Human fibroblasts produce "pregnancy-specific" beta$_1$-glycoprotein *in vitro*. *Am. J. Obstet. Gynecol.*, **134**, 734.

TATARINOV, Y. S., FALALEEVA, D. M., KALASHNIKOV, V. V. & TOLOKNOV, B. O. (1976) Immunofluorescent localisation of human pregnancy-specific $\beta$-globulin in placenta and chorioepithelioma. *Nature*, **260**, 263.

WAHLSTRÖM, T., LINDER, E. & SAKSELA, E. (1973) Glia-specific antigens in cell cultures from rabbit brain, human foetal and adult brain and gliomas. *Acta Pathol. Microbiol. Scand.* (Sect. B), **81**, 768.

WAHLSTRÖM, T., LINDER, E., SAKSELA, E. & WESTERMARK, B. (1974) Tumor-specific membrane antigens in established cell lines from gliomas. *Cancer*, **34**, 274.

WAHLSTRÖM, T. & SEPPÄLÄ, M. (1979) Luteinizing hormone-releasing factor-like immunoreactivity in islet cells and insulomas of the human pancreas. *Int. J. Cancer*, **24**, 744.